# Assessing the Real-time Influence of Racism-Related Stress and Suicidality Among Black Men: Protocol for an Ecological Momentary Assessment Study

**DOI:** 10.2196/31241

**Published:** 2021-10-20

**Authors:** Leslie Adams, Godwin Igbinedion, Aubrey DeVinney, Enoch Azasu, Paul Nestadt, Johannes Thrul, Sean Joe

**Affiliations:** 1 Department of Mental Health Johns Hopkins Bloomberg School of Public Health Johns Hopkins University Baltimore, MD United States; 2 Washington University at St. Louis St. Louis, MO United States; 3 Centre for Alcohol Policy Research Melbourne Australia; 4 Sidney Kimmel Comprehensive Cancer Center Johns Hopkins University Baltimore, MD United States

**Keywords:** Black men, suicide, racism, ecological momentary assessment

## Abstract

**Background:**

Suicide is the third leading cause of death among Black adults aged 18-35 years. Although men represent a majority of suicide deaths among Black adults, less is known regarding the extent to which unique cultural stressors, such as racism-related stress (eg, racial discrimination), are salient in exacerbating suicide risk among Black men. Moreover, few studies examine the daily influence of racism-related stressors on suicide outcomes using real-time smartphone-based approaches. Smartphone-based mobile health approaches using ecological momentary assessments (EMA) provide an opportunity to assess and characterize racism-related stressors as a culturally sensitive suicide risk factor among Black young adult men.

**Objective:**

The goal of this study is to describe a protocol development process that aims to capture real-time racism-related stressors and suicide outcomes using a smartphone-based EMA platform (MetricWire).

**Methods:**

Guided by the Interpersonal Theory of Suicide (ITS), we developed a brief EMA protocol using a multiphased approach. First, we conducted a literature review to identify brief measures previously used in EMA studies, with special emphasis on studies including Black participants. The identified measures were then shortened to items with the highest construct validity (eg, factor loadings) and revised to reflect momentary or daily frequency. Feasibility and acceptability of the study protocol will be assessed using self-report survey and qualitative responses. To protect participants from harm, a three-tier safety protocol was developed to identify participants with moderate, elevated, and acute risk based on EMA survey response to trigger outreach by the study coordinator.

**Results:**

The final EMA protocol, which will be completed over a 7-day period, is comprised of 15 questions administered 4 times per day and a daily questionnaire of 22 items related to sleep-related impairment and disruption, as well as racism-related stress. Study recruitment is currently underway. We anticipate the study will be completed in February 2023. Dissemination will be conducted through peer-reviewed publications and conference presentations.

**Conclusions:**

This protocol will address gaps in our understanding of Black men’s suicide outcomes in the social contexts that they regularly navigate and will clarify the temporal role of racism-related stressors that influence suicidal outcomes.

**International Registered Report Identifier (IRRID):**

PRR1-10.2196/31241

## Introduction

In recent years, suicide completion rates among Black Americans have increased significantly [1-4]. According to the Centers for Disease Control and Prevention, in 2018, suicide emerged as the third-leading cause of death among Black adults aged 18-35 years [5]. Among Black suicide decedents, men comprise the majority (81%) [5]. In response to rising rates of suicide completion, researchers and policy makers have identified the timely recognition and mitigation of suicide risk factors among Black young adult individuals as an emerging public health priority [3,4,6]. Although previous research has identified potential therapeutic approaches for Black males at risk of suicide [7], few studies have identified distinguishable risk factors occurring in Black men’s daily social environment that may exacerbate suicidal thoughts and behaviors. To address this knowledge gap, cultural factors that affect the lived experience of Black men warrant further exploration.

Scholars assert that, among Black Americans, racial discrimination is a chronic stressor that may be more likely to result in a lower quality of life and higher psychological distress compared to their White counterparts [8-10]. Previous studies show that racism-related stressors are directly linked to poorer mental health outcomes, and are also specifically associated with fatal and nonfatal suicide outcomes in Black populations [11,12]. Goodwill and colleagues [10] found that, when compared to other sources of everyday discrimination (eg, generalized or attributed to other marginalized status such as gender or age), everyday race-based discrimination was the only type of discrimination that was significantly associated with increased rates of depressive symptoms and suicidal ideation. Although past studies examined frequent exposure to racial discrimination and its association with suicidal behaviors, authors of these studies suggest that this stressor operates dynamically over time as a function of the social environment that Black men regularly navigate. Indeed, racism is embedded in our society such that it creates dynamic subsystems that constantly reinforce one another [11,12], and thus cannot be captured at a single time point using a cross-sectional design alone. Thus, methodological approaches for examining dynamic shifts in racism-related stress exposures and suicide outcomes are needed.

The Interpersonal Theory of Suicide (ITS), developed by Thomas Joiner [13,14], has been a frequently applied framework to understand the proximal risk factors that precede suicidal behavior. This theory proposes that suicidal desire manifests from two interpersonal constructs—thwarted belongingness and perceived burdensomeness—with both being mediated by hopelessness. Additionally, ITS posits that the capability to engage in suicidal behavior is distinct from the desire to engage in that behavior [14]. To date, this model has not been applied extensively to Black men and does not adequately incorporate racism-related stressors as a potential mechanism in this behavioral process. One recent study applying the ITS model among African Americans found that hope moderated the relationship between thwarted belongingness and suicidal ideation [15]. Another study highlighted the relationship between racism and hopelessness and found that daily racial discrimination resulted in increased hopelessness among Black participants compared to other participants of varied racial and ethnic backgrounds that experience feelings of hopelessness [16]. Collectively, these studies identify racial discrimination as a unique antecedent to key risk factors related to suicide among Black Americans. Guided by these extant studies, additional research is needed to further test these explanatory mechanisms through more robust methodological designs that capture the dynamic, real-time, and longitudinal nature of racism-related stressors in the suicide experience.

Ecological momentary assessment (EMA) can be used to assess health information in real time, making this an effective approach to assess dynamic phenomena such as racism-related stressors. EMAs are used to repeatedly assess a sample’s dynamic changes in behavior and experience in real time [17,18]. EMA approaches are useful for this research as they can be accessed conveniently through smartphones, which broadens the scope of this approach’s applicability compared to paper-and-pencil or computer-assisted surveys. EMA also has noted advantages compared to other cross-sectional and longitudinal methods, such as minimizing recall bias and obtaining more accurate data, as these assessments are conducted in the subject’s natural environment where they would feel most comfortable [17,18].

Despite technological advances in smartphone-based research and EMA studies, few studies have extended this approach to assess suicide outcomes in historically racialized populations such as Black men. To address these evidence gaps, the goal of our study is to develop a theory-informed EMA protocol for suitable use with a psychiatric sample of Black men aged 18-35 years. By integrating EMAs and smartphone technology, our findings will allow researchers to further understand how racism-related stressors may play a role in young Black men’s daily life experiences and suicidal ideation and behaviors. Findings from this study will extend our understanding of the time-varying role of racism-related stressors beyond extant cross-sectional research and assess proximal factors of suicidal thought and behavior in real time.

## Methods

### Project Overview

**Figure 1:**
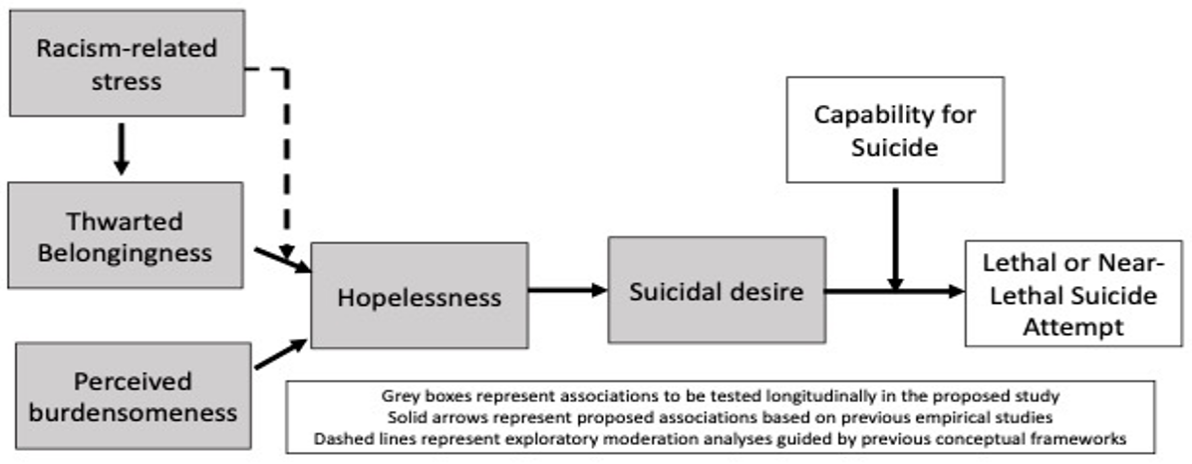
Adapted conceptual framework guided by Interpersonal Theory of Suicide.

The EMA study is part of a 2-year mixed methods investigation of the relationship between racism-related stress and suicidal thoughts and behaviors among Black young adult men aged 18-35 years. The larger study employs an exploratory sequential mixed methods design to adequately explore the phenomenon of rising suicide rates among Black men and integrate qualitative themes into more robust quantitative data collection processes in our EMA study at later phases [19]. In this study, we present our a priori EMA protocol development aimed at evaluating real-time assessments of suicidology among Black men. Eligible participants will complete a 7-day EMA procedure, whereby they self-report momentary suicidal thoughts and behaviors and proximal risk factors derived from our adapted ITS framework (Figure 1) using a smartphone-based app. We will also assess everyday experiences of racism-related stress and sleep patterns.

### Target Population and Eligibility

Eligible participants will meet the following inclusion criteria: (1) be aged 18-35 years; (2) identify as Black or African American; (3) have a past history of suicidal thoughts or behavior; (4) be able to speak, read, and understand English sufficiently well to complete the procedures of the study; (5) have a smartphone; and (6) have an outpatient mental health provider. We will exclude those who have been diagnosed with an active psychotic disorder, those with cognitive deficits or a medical condition that precludes full understanding of study materials, and those who are currently incarcerated.

### Sample Size and Recruitment Procedures

Our intended study sample for this pilot study is 50 participants, which will yield initial feasibility data for the recruitment and retention approach of our study protocol for future scalable projects. The sample size was derived from previous EMA studies focused on suicide assessment in psychiatric populations [20-22]. For this sample size and study duration, we conservatively anticipate an 80% compliance rate, defined as completion of 23 surveys over the 7-day period.

We will identify eligible participants using two purposive sampling approaches. First, we will elicit direct referrals from Johns Hopkins Hospital outpatient psychiatrists, psychiatric nurses, and clinical social workers treating patients who fit the eligibility criteria. Additionally, we will use a clinical research recruiting service (sponsored by the Johns Hopkins Institute for Clinical and Translational Research) that identifies eligible participants in EPIC electronic health record databases. We will then provide recruitment information to active patients via MyChart, a web-based patient portal that provides patients with their personal health information and medical history and allows for communication between the patient and their health care provider or health care system. Eligible participants from both recruitment approaches will be referred to contact the study coordinator, who will then verify eligibility using an online screening survey. Once eligibility is confirmed, the coordinator will schedule a brief telephone discussion with the participant to provide additional information regarding the study and initiate the informed consent and enrollment process via REDCap.

### Baseline Survey

Enrolled participants will be asked to complete a brief baseline assessment via REDCap. The survey will assess demographic characteristics, such as sexual orientation, education, and socioeconomic status. We will also include validated psychosocial measures associated with affective, gender, and race-specific factors associated with suicidal thoughts and behaviors, such as anger, sadness, attributional style, and racial identity. A complete list of baseline measures is presented in Table 1. Following completion, participants will receive a 15-minute overview of the MetricWire smartphone app and EMA questions via phone or Zoom call with the study coordinator. During this time, the study coordinator will schedule prompt times during regular waking hours (eg, 8AM to 10PM), schedule a follow-up time for the participant’s exit interview, and record the contact information of the participant’s outpatient mental health provider, when available, to implement into our safety protocol.

**Table 1:** Overview of baseline, ecological momentary assessment, and daily diary survey measures.

Variable	Baseline	Ecological momentary assessment (4 times per day)	Daily diary (once per day)
Age	✓		
Sexual orientation	✓		
Marital status	✓		
Employment status	✓		
Education status	✓		
Racial identity (Multidimensional Inventory of Black Identity [MIBI]) [23]	✓		
Anger (Dimensions of Anger Reactions-5 item [DAR-5]) [24]	✓		
Affective emotional states (Positive Affect and Negative Affect Schedule-Expanded Form [PANAS-X]) [25]	✓		
Emotional regulation (Emotional Regulation Questionnaire [ERQ]) [26]	✓		
Generalized social anxiety (Mini Social Phobia Inventory [Mini-SPIN]) [27]	✓		
Happiness (Pemberton Happiness Index [PHI]) [28]	✓		
Callousness (Inventory of Callous-Unemotional Traits [ICU]) [29]	✓		
Grit-S scale [30]	✓		
Psychache [31]	✓		
Suicidal thoughts and behavior (Columbia Suicide and Severity Rating Scale [C-SSRS]) [32]	✓	✓	
Hopelessness (Beck Hopelessness Scale [BHS]) [33]		✓	
Major depressive disorder (Patient Health Questionnaire-2 item [PHQ-2]) [34]		✓	
Perceived burdensomeness (Interpersonal Needs Questionnaire-Perceived Burdensomeness Subscale [INQ-PB]) [35]		✓	
Racism-related stress (Everyday Discrimination Scale [EDS]) [9]			✓
Capability of suicide (Acquired Capability for Suicide Scale [ACSS]) [36]		✓	
Thwarted belonginess (Interpersonal Needs Questionnaire-Thwarted Belongingness Subscale [INQ-TB]) [35]		✓	
Sleep-related impairment (Patient-Reported Outcomes Measurement Information System [PROMIS]) [37]			✓
Sleep disturbance (Patient-Reported Outcomes Measurement Information System [PROMIS]) [37]			✓

### Data Collection Procedures

**Figure 2:**
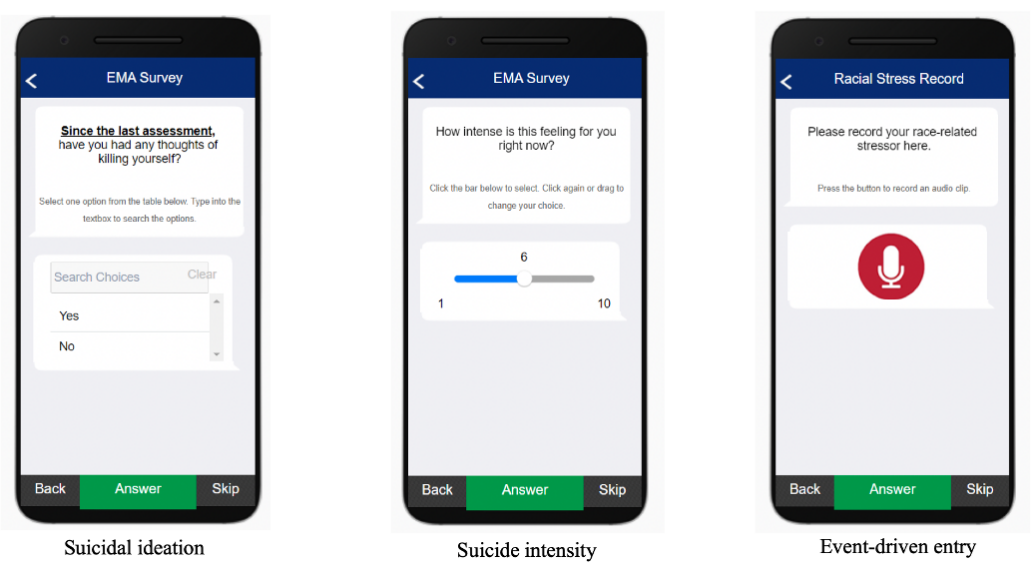
Select screenshots from the EMA study smartphone platform (MetricWire). EMA: ecological momentary assessment.

After completion of the baseline survey, participants will be asked to download the MetricWire app onto their personal smartphone for the study duration. The MetricWire app is available for both iOS and Android smartphone platforms at no cost in the Apple App Store or Google Play Store, respectively. Examples of the user interface of the MetricWire app are presented in Figure 2.

The MetricWire app will deliver EMA surveys at four semirandom timepoints per day during participant waking hours, which will be determined at baseline. Based on the participant-defined waking hours, the first prompt will begin 30 minutes after waking time. Each of the four daily prompts will have three push notification reminders at 20, 40, and 60 minutes after the initial prompt. If the EMA survey is not completed after 60 minutes, the survey will be marked as incomplete. To fully capture instances of racism-related stressors occurring outside of random EMA survey prompts, participants will also have the option to record event-driven entries detailing their daily experiences (Figure 2, right panel). EMA surveys were designed to take no more than 3 minutes to complete to reduce respondent burden. Once per day, we will administer a brief daily diary survey via the MetricWire app to assess everyday experiences that were not determined to occur at momentary instances, including sleep-related impairment and quality, and daily experiences with racism-related stress [9]. At the conclusion of the 7-day data collection period, the study team will conduct a qualitative semistructured exit interview with each participant, and probe the participant on issues related to question difficulty and clarity, revision of question prompts, and overall satisfaction with the study protocol and EMA surveys. We anticipate the semistructured interview to last 30-60 minutes in total. Interviews will be transcribed verbatim and used to refine the EMA study protocol.

### Participant Incentives

Participants will receive $100 in total, incrementally phased throughout the duration of the study to encourage higher EMA survey completion rates. Participants will receive $20 after completing the baseline survey, $20 at the completion of day three, and $20 at the conclusion of the 7-day study duration and baseline interview. Participants will receive an additional $40 if they complete at least 80% of the EMA surveys during the study period.

### Safety Protocols

Previous research has demonstrated that repeated measures of suicide do not illicit suicidal thoughts or behaviors [38]. However, considering the high risk for suicide among our target sample, and to reduce the potential harm associated with repeated questions about suicide, our team will implement several safety protocols to support the mental well-being of participants enrolled in our study. Upon enrollment, participants will receive a document outlining local and national mental health resources, including suicide crisis hotlines. Additionally, all research personnel interacting with participants will be trained in psychological first aid to assist in identifying any mental health needs that arise throughout the study. EMA responses by participants will be monitored daily by the study coordinator and discussed during weekly research team meetings. Risk will be categorized in our EMA protocol using a three-tier risk designation. Moderate risk, which is defined as any suicidal ideations (“Have you had thoughts of killing yourself?”) since the last assessment, but without any plan or intent, will result in a notification to the participant guiding them to the appropriate services and urging them to seek support with their mental health outpatient provider’s phone number. Elevated risk, defined as suicidal ideation with intent or a plan within the last 24 hours (“Have you planned out how you would do it?” and/or “When you thought about killing yourself, did you think that this was something you might actually do?”), will result in the same notification given to participants with moderate risk followed by a notification to their mental health outpatient provider. In this risk category, we will also offer to contact a local crisis response hotline on their behalf. Acute risk, defined as suicidal ideation with an action since the last assessment (“Did you do anything to try to kill yourself?”), will result in an immediate call to the local crisis response hotline made by a study team member on the participant’s behalf. Safety risk alerts are programmed in MetricWire to notify the study coordinator, who will then immediately inform outpatient providers of patient responses for follow-up.

### Selection of EMA Measures

To discuss the most appropriate EMA survey measures, authors (LBA, GI, EA, AD, and SJ) met weekly from November 2020 to March 2021. Based on previous EMA feasibility studies, our goal was for the duration and length of our EMA survey to last 2-3 minutes, with 20 or fewer items [17,39]. Our weekly meetings focused on measure selection as well as the suitability of each measure for the baseline survey, the EMA questionnaire, or other aspects of data collection (eg, exit survey or daily diaries).

We first identified full and brief measures related to ITS derived from our adapted conceptual framework (Figure 1), including thwarted belongingness, perceived burdensomeness, and capability for suicide [35,39,40]. We then conducted a directed literature review of brief validated measures by searching MEDLINE (PubMed) and Google Scholar to identify additional brief measures to include in our surveys. Search strategies for each database were developed in coordination with authors (LBA and SA). This search strategy included terminology related to methods (eg, digital phenotyping, smartphone, EMA), target population (eg, Black, African American), and affective states (eg, anger, fear, and happiness) relevant to our study objectives. Priority was given to studies that focused on or included our target population in the study sample and items that were previously used in EMA studies. Additional studies were also included by examining the reference lists of included studies.

To reduce validated measures into brief EMA items for our survey, we reviewed selected measures to determine their validity and reliability in our target population. We also reviewed factor analysis studies of each measure to determine items in the overall scale that were more closely related to the measured construct, incorporating items with the highest factor loading into our EMA protocol. For instance, to briefly assess hopelessness, we selected items from the Beck Hopelessness Scale with the highest factor loadings in a previous construct validity study, yielding our retention of the items “I feel that things won’t work out” (item 14; 0.872 factor loading) and “I feel there is no use in really trying” (item 16; 0.912 factor loading) [33]. Finally, once items were selected, the wording of the questions was changed to reflect the real-time, momentary timing of the measures (eg, since the last prompt). Our final list of items is presented in Table 2.

**Table 2:** Ecological momentary questionnaire items and response options.

Measure	Items (Since the last prompt...)	Response options
Suicidal thoughts and behaviors	Have you had thoughts of killing yourself? Have you planned out (worked out the details of) how you would do it? When you thought about killing yourself, did you think that this was something you might actually do? Did you do anything to try to kill yourself?	Yes or No
Suicidal intensity	How intense is this feeling for you right now?	Likert scale 1-10
Depression [34]	I have little interest or pleasure in doing things I am feeling down, depressed, or hopeless	Likert scale: 0=Not at all, 1=Some of the time, 2=Most of the time, 3=All of the time
Thwarted belongingness [35]	I feel close to others I feel like I belong	Yes or No
Perceived burdensomeness [25]	I feel that people would be happier without me I feel that right now people would be better off if I was gone	Yes or No
Hopelessness [33]	I feel that things won’t work out I feel there is no use in really trying	True or false
Capability for suicide [40]	I could kill myself if I wanted to. I am very much afraid to die.	Likert scale 1-10

### Acceptability and Feasibility

Feasibility will be assessed by the following: (1) the ratio between the number of people who enroll in the study and the total number of people approached for recruitment and/or who complete a screening survey, (2) participant reports of ease of completing the EMA survey (Likert scale: 1-5), (3) percentage low compliance (defined as 50% or fewer EMA surveys completed during the 7-day period), (4) percentage of safety-related incidents reported to clinical staff (eg, acute suicide risk notifications), and (5) average length of time to complete surveys. Acceptability will be assessed using qualitative responses from semistructured exit interviews at the end of data collection. Participants will also have the option to write free-text responses reflecting upon the acceptability of the study.

### Data Analysis

To address known issues with missingness associated with EMA survey compliance [17], we will include only respondents who completed 50% or more of their EMA surveys. We anticipate that our estimated sample size of 50 participants will yield 1120 completed EMA survey responses (accounting for 80% compliance), 350 daily diary responses, and 50 baseline questionnaire surveys for subsequent cross-sectional and longitudinal analyses. Descriptive statistics will be used to characterize the study population in terms of baseline characteristics and feasibility measures using means, *t* tests, chi-square tests (categorical outcomes), and bivariate correlations (continuous outcomes). Descriptive statistics will be summarized by EMA survey compliance (eg, <50%, ≥50%, and ≥80%). We will compute Cronbach α and item-to-total correlations of baseline survey measures. Differences in survey compliance based on baseline characteristics will also be examined using linear regression.

Our primary analysis will investigate the hypothesized mediation model in Figure 1, which will assess the influence of racism-related stressors on suicide outcomes, through the mediating influence of ITS constructs (eg, thwarted belongingness, perceived burdensomeness, and hopelessness). We will conduct multilevel mediation path models using randomly prompted EMA data in Mplus (version 8.4), accounting for the hierarchical data structure of the repeated EMA signals (level 1: within subject) nested within participants (level 2: between subjects) [41]. We will employ maximum likelihood estimation approaches to account for missing data. Tests for moderation will be conducted by using cross-level interactions in the mediation model. Once significant (*P*<.05) predictive, mediating, and moderating factors are identified, we will extract the following information for each significant variable to sufficiently power future multilevel intervention studies: means, variances, parameter estimates, and patterns of missingness.

Qualitative responses from respondent-driven recordings of racism-related events will be analyzed using an iterative thematic approach [42,43]. Recorded events will be professionally transcribed verbatim. Deidentified transcripts will be uploaded onto Dedoose software for subsequent analysis [44]. Final qualitative themes will enhance our understanding of daily experiences of racism among a psychiatric sample of Black men.

## Results

Research team meetings to select and modify measures for our study resulted in a 15-item EMA survey administered 4 times per day and a 22-item daily diary survey administered at the end of each day. As of July 2021, recruitment for the qualitative phase of our mixed methods study is currently underway. Inclusion of participants for the EMA phase of our study will begin as early as August 2021 and will conclude by December 2022. Complete data collection and analyses are expected to conclude by February 2023. Preliminary results are expected to be disseminated in peer-reviewed journals and presented at national conferences starting in Spring 2022. All phases of the research study have been approved by the Institutional Review Board at Johns Hopkins Bloomberg School of Public Health (#00013672).

## Discussion

### Principal Findings

The anticipated results of the study will inform how racism-related stressors influence both proximal risk factors and suicidal thoughts and behaviors in a psychiatric sample of Black young adult men. The significance of the research provides timely explanatory evidence toward the growing trend of suicide completion among Black men, who comprise the largest percentage of deaths by suicide (81%) within the Black community [5]. The goal of this protocol is twofold: (1) to demonstrate the suitability of EMA methods in assessing real-time momentary changes of suicidality within Black men and (2) to clarify the temporal role of real-time racism-related stressors in the experiences leading to suicidal outcomes. The proposed research is the first to our knowledge to address critical research gaps in suicide research, including the consideration of racism-related stress in the theoretical and empirical application of the ITS. Additionally, our research team plans to leverage the full promise of intensive longitudinal data collection procedures using EMA and daily diary surveys among a within-group sample of Black men, an underrepresented and understudied population in suicide prevention research.

### Limitations

Participant burden, noncompliance, and reactivity to the protocol measures have been cited as potential limitations to EMA and smartphone-based mobile health studies [18,45]. We are encouraged by extant research demonstrating a median response rate of 75% or higher over longer periods among psychiatric patients and young adults [21,46-49]. To encourage steady compliance, participant incentives will be distributed incrementally, which has been done successfully in previous studies [45]. In the event of steady poor compliance and reduced yield of expected observations, we will leverage the following: (1) completed EMA surveys collected at earlier time points (eg, day 1 and day 2 of the 7-day study), (2) the daily diary, and (3) baseline cross-sectional survey responses. This study is also limited in its generalizability to Black men receiving psychiatric care in an academic research hospital in Baltimore, MD. Future studies should consider additional venues and settings to recruit Black men who are not engaged in psychiatric care, including but not limited to social media, advocacy groups, and peer-led and/or community-based organizations.

### Conclusions

Despite these limitations, our proposed study has the potential to more robustly identify and assess the impact of daily racialized stressors among Black men with mental health needs. Study results will provide insights regarding the temporal influence of frequent racism-related stress, which can be clarified further and potentially mitigated in future suicide prevention research across multiple settings. Findings from this study will also generate key hypotheses for future fully powered EMA and intervention research that includes information on missingness patterns, compliance, and parameter estimates of key study variables. In addition to assessing the suitability of EMA approaches to capture daily racialized experiences and proximal suicide outcomes, our findings can be adapted to other ecologically valid contexts that may exacerbate Black young adult men’s mental health outcomes in real time, such as police killings of unarmed Black men and other direct and vicarious experiences of racial trauma [50,51]. Overall, our study will provide important insights to help bridge the gap in research regarding Black men’s suicidality and will serve as a model for future real-time smartphone-based assessments focused on this vulnerable population.

## Appendix

Multimedia Appendix 1 Peer review response from the American Foundation of Suicide Prevention.

## Abbreviations

EMA: ecological momentary assessment
ITS: Interpersonal Theory of Suicide
