# Supplementary material for: Assessing the Real-time Influence of Racism-Related Stress and Suicidality Among Black Men: Protocol for an Ecological Momentary Assessment Study
Source: JMIR Res Protoc. 2021 Oct 20;10(10):e31241. doi: 10.2196/31241 (PMC8567147; doi:10.2196/31241)
Supplement: Multimedia Appendix 1 [file resprot_v10i10e31241_app1.pdf]

Response to Reviewers for:
"Real-time assessments of suicidality among Black men: a mixed methods approach"
PI: Leslie Adams, PhD, MPH
Grant number: YIG-0-001-19

*Excerpted reviewer comments appear in ***bold italics***, responses appear in plain text

---

We thank the AFSP YIG reviewers for their thoughtful consideration of this applications. We have provided responses to the reviewer critiques below to describe improvements during the AFSP YIG funding period:

1. Consider including all facets of the model and potential factors that may contribute to the model or impact.

Thank you for your comment. Joiner's interpersonal theory of suicide (ITS), which posits interactive roles of perceived burdensomeness, thwarted belonging, and acquired capacity for suicide in driving suicidal risk, has strong predictive power in adults, but has not yet been tested temporally to assess suicide risk among Black men. Additionally, extant research has shown that race-related stressors, such as racial discrimination and microaggressions, may have a significant effect on suicidality among Black men,[1–3] but the temporal, reactive effect of this stressor has not been fully explored in suicide prevention research. <u>To address this reviewer critique</u> and to expand the applicability of this framework, we plan to use an apriori approach in developing our measures for the ecological momentary assessment adaptation proposed in Aim 2 that includes most components of the ITS framework (Figure 1). Table 1 illustrates additional consideration of the ITS framework and associated validated measures that will be included in our ecological momentary assessment (EMA) smartphone application adaptation (Aim 2). Additionally, we will add a hypothesis to our overall study that those who report active and passive suicidal ideation, when measured via the EMA application, will increase the likelihood of reporting enhanced capability for suicide. In concert with expanding the measurable aspects of the ITS model, we have enhanced our data safety and monitoring protocol to include a two-tier suicide risk identification system in our EMA protocol, additional training of study personal in Mental Health First Aid, and contact information of participants in the event of referring them to acute mental health crisis resources. Along with this response, we have attached a draft protection of human subjects protocol that will be included in our forthcoming IRB application to Johns Hopkins University.

Figure 1: Conceptual Framework of AFSP study guided by Interpersonal Theory of Suicide (ITS)

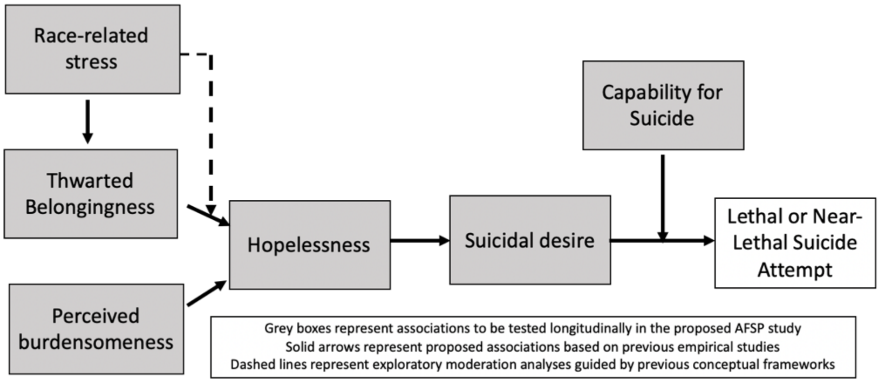

**Table 1: Overview of apriori random-prompt measures and sample items for EMA adaptation based on adapted ITS model (Aim 2)**				
**ITS construct**	**Scale name**	**Citation**	**# of items**	**Example item**
Passive suicidal ideation (PSI)	Paykel Suicide scale (PSS); Hamilton Rating Scale for Depression (HRSD)	Paykel et al., 1974; Hamilton, 1960[4,5]	5 (PSS); 1(HRSD)	Life is not worth living for me.
Active suicidal ideation (ASI)	Modified Scale for Suicidal Ideation; (MSSI) Beck Scale for Suicidal Ideation (BSSI);	Miller et al. 1986; Beck & Steer, 1991, 1997[6–8]	18 (MSSI); 21(BSSI)	I want to die.
Perceived Burdensomeness	Interpersonal Needs Questionnaire-Perceived Belongness subscale (INQ-PB)	Joiner, 2009; Van Orden, 2008[9,10]	6	I feel like a burden for others
Thwarted Belongness	Interpersonal Needs Questionnaire-Thwarted Belongingness Subscale (INQ-TB)	Joiner, 2009; Van Orden, 2008[,7]	9	I feel like I do not belong
Hopelessness	Beck Hopelessness Scale (BHS)	Beck & Steer 1989[11]	20	My future seems dark to me.
Capability for Suicide	Acquired Capability for Suicide scale (ACSS)	Van Orden et al, 2008[10]	20	The pain involved in dying frightens me
General stress	Daily Inventory of Stressful Events (DISE)	Almeida et al., 2002[12]	7	Did you have an argument or disagreement with someone today?
Race-related Stress	Everyday Discrimination Scale (EDS)	Williams et al, 1997[13]	10	I am called names or insulted (due to my race)
Negative Affect	Positive and Negative Affect Scale (PANAS)	Watson et al, 1988[14]	11	I am lonely

2. Some factors affecting risk may not be related to race. Also consider diagnosis and genetics.
Thank you for this comment. We agree with reviewer's comments that additional suicide risk factors may include clinical diagnoses, particularly related to DSM-V disorders, and genetic influence. Findings from this award will be positioned within the full context of suicide risk factors, which includes the recent studies related to genetics research, which finds that family history of suicidal behavior and psychopathology increases suicide risk.[15,16] These findings also illustrate the importance of genetic variation and suicide risk.[16] Additionally, research shows that prior diagnoses related to depression, bipolar disorder, schizophrenia, borderline or antisocial personality disorders; conduct disorder; psychotic disorders or symptoms; substance abuse disorders may be predictive of future suicidal behavior.[17] Given the real-time, dynamic nature of ecological momentary assessment technology, we may not be able to identify fluctuations in symptoms related to the aforementioned DSM-V diagnoses. To address reviewer critique regarding diagnoses and genetic factors, we plan to also obtain informed consent during the recruitment process to access EMA participants' electronic health record data in the Johns Hopkins University hospital system. From this data, we will be able to query a broad range of clinical diagnoses as well as past family history of suicidal behavior and psychopathology as baseline characteristics that may place participants at increased risk of suicidal ideation.

3. Work closely with your mentorship team from the beginning.
Thank you for this comment. I am enthusiastic about working with my mentorship team, as stated in the initial proposal, which includes Drs. Sean Joe (Washington University at St. Louis), Benjamin Lê Cook (Harvard Medical School/Cambridge Health Alliance), and additional EMA technical consulting from Dr. Todd Reid (Harvard T.H. Chan School of Public Health) and Johannes Thrul (Johns Hopkins Bloomberg School of Public Health). Dr. Joe and I have been working collaboratively on projects and manuscripts related to Black male suicidality since April 2019. For this award, Dr. Joe will provide specific content expertise in suicide research in Black American populations, to advise me toward independence during the YIG study period. To address this reviewer critique, Dr. Joe and I are meeting weekly to discuss progress on the study and to cultivate my knowledge towards Black men's suicide research. We have been adhering to this weekly meeting schedule, via Zoom, since June 2020 and have future meetings scheduled at this frequency until March 2021. Biweekly meetings with Dr. Benjamin Lê Cook and Todd Reid will begin at the beginning of the study period. The meetings will be guided by the study workplan and timeline below:

**Initial Grant Timeline**	Year 1												Year 2											
Activity/Process	1	2	3	4	5	6	7	8	9	10	11	12	13	14	15	16	17	18	19	20	21	22	23	24
**Research Activities**																								
IRB approval and modifications	■										■					■								
Hire Graduate Research Assistant	■	■																						
Aim 1: Refine moderator's guide	■	■																						
Aim 1: Recruit participants (n=20; recruit 5 participants each month)			■	■	■	■	■																	
Aim 1: Conduct Focus Groups			■	■	■	■	■																	
Aim 1: Thematic Analysis							■	■	■	■	■													
Aim 1: Member Checking										■	■	■												
Aim 2: Map qualitative themes to ecological momentary assessment (EMA) protocol											■	■	■											
Aim 2: Revise EMA protocol using joint display tables													■	■	■									
Aim 3: Recruit participants for EMA study (n=30; recruit 10 participants each month)															■	■	■							
Aim 3: Conduct EMA study															■	■	■	■	■	■	■			
Aim 3 Longitudinal Analysis Assessing significant predictors of suicidality																				■	■	■	■	■
**Career Development**																								
Develop and implement directed reading list w/ Dr. Sean Joe	■	■	■	■	■	■																		
Manuscript development			■	■	■	■	■	■	■	■	■	■	■	■	■	■	■	■	■	■	■	■	■	■
U-M Mixed Methods Workshop (Oct 2021)													■											

References

1. Goodwill JR, Taylor RJ, Watkins DC. Everyday Discrimination, Depressive Symptoms, and Suicide Ideation Among African American Men. *Arch Suicide Res*. Published online October 10, 2019:1-20. doi:10.1080/13811118.2019.1660287

2. Arshanapally S, Werner KB, Sartor CE, Bucholz KK. The Association Between Racial Discrimination and Suicidality among African-American Adolescents and Young Adults. *Arch Suicide Res*. 2017;22(4):584-595. doi:10.1080/13811118.2017.1387207

3. Walker R, Francis D, Brody G, Simons R, Cutrona C, Gibbons F. A Longitudinal Study of Racial Discrimination and Risk for Death Ideation in African American Youth. *Suicide and Life-Threatening Behavior*. 2017;47(1):86-102. doi:10.1111/sltb.12251

4. Paykel ES, Myers JK, Lindenthal JJ, Tanner J. Suicidal feelings in the general population: a prevalence study. *Br J Psychiatry*. 1974;124(0):460-469. doi:10.1192/bjp.124.5.460

5. Hamilton M. A RATING SCALE FOR DEPRESSION. *J Neurol Neurosurg Psychiatry*. 1960;23(1):56-62.

6. Miller IW, Norman WH, Bishop SB, Dow MG. The Modified Scale for Suicidal Ideation: reliability and validity. *J Consult Clin Psychol*. 1986;54(5):724-725. doi:10.1037//0022-006x.54.5.724

7. Beck AT, Steer RA. *BSI, Beck Scale for Suicide Ideation : Manual*. Psychological Corp. ; Harcourt Brace Jovanovich; 1991.

8. Beck AT, Brown GK, Steer RA. Psychometric characteristics of the Scale for Suicide Ideation with psychiatric outpatients. *Behaviour research and therapy*. 1997;35(11):1039-1046.

9. Van Orden KA, Witte TK, Cukrowicz KC, Braithwaite SR, Selby EA, Joiner Jr TE. The interpersonal theory of suicide. *Psychological review*. 2010;117(2):575.

10. Van Orden KA, Witte TK, Gordon KH, Bender TW, Joiner TE. Suicidal desire and the capability for suicide: tests of the interpersonal-psychological theory of suicidal behavior among adults. *J Consult Clin Psychol*. 2008;76(1):72-83. doi:10.1037/0022-006X.76.1.72

11. Beck AT, Brown G, Steer RA. Prediction of eventual suicide in psychiatric inpatients by clinical ratings of hopelessness. *Journal of consulting and clinical psychology*. 1989;57(2):309.

12. Almeida DM, Wethington E, Kessler RC. The daily inventory of stressful events: An interview-based approach for measuring daily stressors. *Assessment*. 2002;9(1):41-55.

13. Williams DR, Yan Yu, Jackson JS, Anderson NB. Racial Differences in Physical and Mental Health: Socio-economic Status, Stress and Discrimination. *J Health Psychol*. 1997;2(3):335-351. doi:10.1177/135910539700200305

14. Watson D, Clark LA, Tellegen A. Development and validation of brief measures of positive and negative affect: the PANAS scales. *Journal of personality and social psychology*. 1988;54(6):1063.

15. Fazel S, Runeson B. Suicide. *New England Journal of Medicine*. 2020;382(3):266-274. doi:10.1056/NEJMra1902944

16. Franklin JC, Ribeiro JD, Fox KR, et al. Risk factors for suicidal thoughts and behaviors: A meta-analysis of 50 years of research. *Psychol Bull*. 2017;143(2):187-232. doi:10.1037/bul0000084

17. Risk factors and warning signs. American Foundation for Suicide Prevention. Published December 25, 2019. Accessed June 22, 2020. https://afsp.org/risk-factors-and-warning-signs#suicide-risk-factors
